# Acoustic source localization with microphone arrays for remote noise monitoring in an Intensive Care Unit

**DOI:** 10.1016/j.apacoust.2018.04.019

**Published:** 2018-10

**Authors:** Markus Müller-Trapet, Jordan Cheer, Filippo Maria Fazi, Julie Darbyshire, J. Duncan Young

**Affiliations:** a Institute of Sound and Vibration Research, University of Southampton, University Rd, Southampton SO17 1BJ, United Kingdom; b Nuffield Department of Clinical Neurosciences, Level 6, West Wing, John Radcliffe Hospital, Oxford OX3 9DU, United Kingdom

**Keywords:** Noise source localization, Microphone arrays, Intensive Care Unit

## Abstract

An approach is described to apply spatial filtering with microphone arrays to localize acoustic sources in an Intensive Care Unit (ICU). This is done to obtain more detailed information about disturbing noise sources in the ICU with the ultimate goal of facilitating the reduction of the overall background noise level, which could potentially improve the patients’ experience and reduce the time needed for recovery. This paper gives a practical description of the system, including the audio hardware setup as well as the design choices for the microphone arrays. Additionally, the necessary signal processing steps required to produce meaningful data are explained, focusing on a novel clustering approach that enables an automatic evaluation of the spatial filtering results. This approach allows the data to be presented to the nursing staff in a way that enables them to act on the results produced by the system.

## 1 Introduction

High noise levels in Intensive Care Units (ICUs) have been reported as a possible contributing factor to patients’ poor physiological recovery [1]. It is thought that these high noise levels can increase the risk of disturbed sleep patterns, hallucinations and periods of delirium [2], [3]. While the World Health Organization (WHO) recommends that sound levels in patient areas should remain below 40dB(A) [4], this limit is often exceeded in ICU environments [5], [6].

A number of strategies have been attempted to reduce sound levels in ICUs. Building design and materials [7], [8], reducing patients’ perception of noise with earplugs or headphones [9], [10] and staff education [11], have all shown variable effectiveness, as has the manipulation of alarm thresholds and volumes [12]. A recent review of noise-reducing measures comes to the conclusion that when patients experience lower noise levels through the use of earplugs, the risk of delirium can be reduced [13].

To better identify the contributing noise sources and explore ways of reducing them, a more detailed understanding of the distribution of acoustic sources over time and space is necessary. Currently, studies use individual sound level meters at discrete positions in the ICU to record the level variation over time [14]. While this offers insight into the periods over which the overall noise activity is higher, it cannot provide information about the distribution of specific sources in time or space. An additional problem is that in order to obtain representative data, sound level meters have to be installed close to the patients’ beds. This often leads to spurious unrealistically high peak levels when staff come into contact with the audio equipment or the supporting structure.

A solution to the problem of using local sound level meters lies in the application of spatial filtering methods such as beamforming to discriminate acoustic sources in space from a remote location [15]. The use of array signal processing methods allows the ICU environment to be scanned for sources with an (almost) arbitrary resolution in a non-intrusive way.

Microphone arrays in combination with spatial filtering methods have found widespread usage in recent years. Typical scenarios where beamforming is used include industrial and environmental noise source identification as well as automotive and aeroacoustic applications [16], [17], [18], [19], [20]. In most of these cases, a manual evaluation of the results in terms of source maps per third-octave band is usually necessary, hence requiring an expert to make sense of the data. Automatic source localization is a problem often encountered in speech signal processing [21], [22], but the approaches are not always generally applicable because of underlying assumptions about the array geometry or the number and type of source signals.

In this paper, a microphone array system designed for the task of automatic source localization is presented and described in detail, with a specific focus on the array signal processing. A modified formulation of the standard Delay-and-Sum beamforming algorithm is presented to increase the computational performance. A deconvolution algorithm usually employed for aeroacoustic measurements is applied here in the context of the ICU environment, leading to increased spatial selectivity. For an automatic evaluation of the beamforming result, a novel clustering approach is described in detail, enabling a display of the results that is easy to understand for non-acousticians.

The outline of the paper is as follows: in Section 2 the setup of the audio hardware of the array system is described. The design of the microphone array is presented in Section 3. The beamforming strategies including the data clustering approach are described in detail in Section 4 together with simulated example data. In Section 5 selected results of measurements made with the system installed in the adult ICU of the John Radcliffe Hospital in Oxford are shown. The paper finishes with concluding remarks in Section 6.

The descriptions in this paper will be useful for replicating the presented array system, and the clustering approach helps to automatically evaluate the result of beamforming calculations and make it presentable to non-technical personnel. Practical considerations for the array system as well as the restrictions imposed by the environment are addressed specifically.

## 2 Hardware setup

**Fig. 1:**
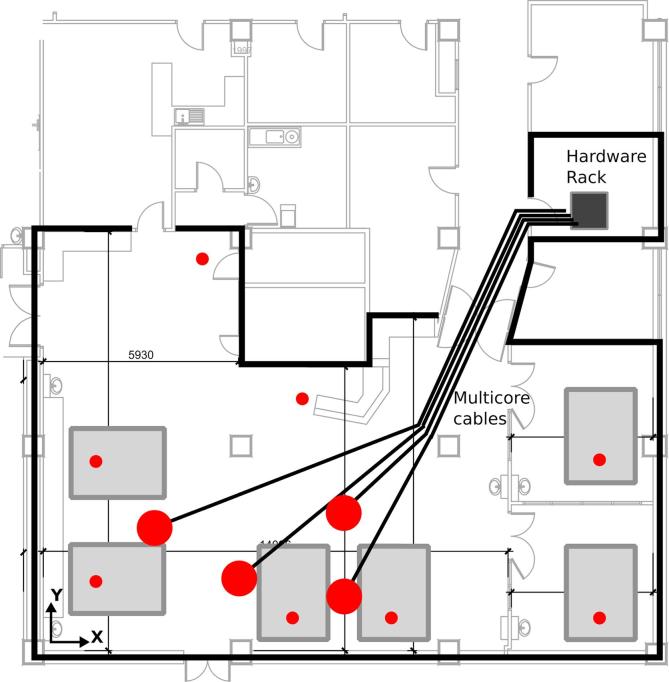
Schematic of the floor plan of the ICU at John Radcliffe Hospital including the location of the beds (grey squares), the microphones (arrays: large red circles; individual: small red circles), the multi-core cables (black lines) as well as the hardware rack. (For interpretation of the references to colour in this figure legend, the reader is referred to the web version of this article.)

To provide insight into the practical setup, a general plan of the ICU environment and the location of the hardware is shown by a schematic diagram in Fig. 1. This includes the location of the beds, microphones and cables.

**Fig. 2:**
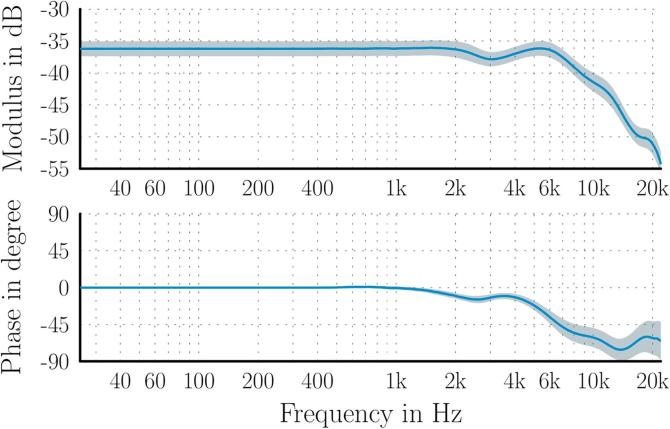
Microphone sensitivity in dB re V/Pa: average result, with standard deviation across all microphones represented by shaded region.

As a cost-effective means to capture audio signals, electret microphones were used. To obtain the best result under the circumstances prescribed by the application, two steps had to be taken. Firstly, the microphone capsules were connected with an electric circuit, which lowers the phantom voltage of 48V down to the supply voltage of approximately 4V, enables longer cables between the microphones and the pre-amplifiers and provides a balanced signal for improved interference rejection. Secondly, all microphones were calibrated over the entire frequency range to match the level and phase response for an optimum beamforming result. It has been shown that the phase response has to be matched for all array microphones to obtain beamforming results of high quality [23]. The broadband calibration was carried out in the small anechoic chamber at the ISVR by measuring the frequency response of a Genelec 8010A [24] with each microphone and comparing this to the response measured with a *Brüel* & *Kjær* $\frac{1}{2}$” free-field measurement microphone with a flat frequency response. The average sensitivity response in dB re V/Pa is plotted in terms of the modulus and phase as a function of frequency in Fig. 2. The shaded area represents the standard deviation across all microphones. The calibrated responses were combined with the measured pre-amplifier gains and inverted to obtain equalization filters that are applied to the audio input data in the frequency-domain to convert digital signal levels into sound pressure levels before any further processing takes place.

To avoid spending time developing bespoke hardware, off-the-shelf audio equipment was chosen to provide the microphone pre-amplifiers and digital conversion. To process the microphone signals and provide phantom power, a total of nine Focusrite OctoPre MKII [25] were used, each converting the input voltage of eight microphones into a digital ADAT stream. The eight ADAT streams for the 64 array microphones were then combined into one MADI stream with the RME ADI-648 [26]. For an additional eight microphones distributed in the ICU, a second MADI stream was provided by the Ferrofish A16-MKII [27]. Both MADI inputs were fed to the RME MADI FX sound card [28] to provide digital audio for further processing.

The connection between the hardware rack and the microphones was achieved with five 16-channel multi-core cables of 30m length each. Four of these cables were used to connect to the four sub-arrays (see Section 3) and the fifth combined the signals from the eight individual microphones distributed in the ICU (see the schematic in Fig. 1).

## 3 Array design

As the microphone system was not supposed to interfere in any way with the daily operation of the medical personnel or the patients, the ceiling was chosen as the location of the microphones. This means that a planar array configuration had to be used. Due to the large spatial extent of the ICU (approximately 12m×8m), four clusters of 16 microphones were distributed throughout the ICU to cover all of the patients’ beds (see Fig. 1). For an additional, separate level monitoring, eight individual microphones were also installed in the ceiling above the beds and the nurses’ station. The individual microphones were used for stationary level monitoring above the beds, including beds in two separate rooms that cannot be covered by the array system.

**Fig. 3:**
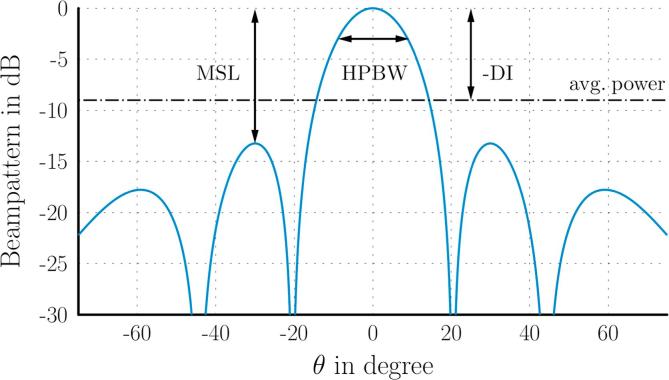
Beampattern for a linear array, and array performance parameters.

For convenient installation, each of the four array clusters of 16 microphones was fitted into a single ceiling tile, with approximate dimensions of 0.6m×0.6m. This – together with the number of microphones – was the restriction for the design of the array configuration. The array performance was measured with typical parameters based on the beampattern of the array [29, Section 2.4.1]: the Half-Power Beamwidth (HPBW) in degrees as a measure of spatial discrimination; the Maximum Sidelobe Level (MSL) in dB as a measure of the dynamic range of the beamforming result; and the Directivity Index (DI) in dB as a measure of the spatial focusing (see the plot in Fig. 3 explaining the parameters for the beampattern of a linear array). For the data presented here, the performance parameters were evaluated on a hemi-spherical grid with a radius of 1m and 1deg angular resolution. The results were chosen as the worst-case across all directions, to obtain a lower limit of the performance.

Of the many possible planar array geometries – such as the regular grid, cross grid, logarithmic spiral array, circular array and random array – the circular array and logarithmic spiral array were regarded as relevant in this project. These two array geometries yield a spatial selectivity that does not vary too much with the azimuthal steering angle and are relatively easy to build. Several combinations of array radii and distribution of sensors were tested for each of these two array types and rated according to the aforementioned performance parameters.

**Fig. 4:**
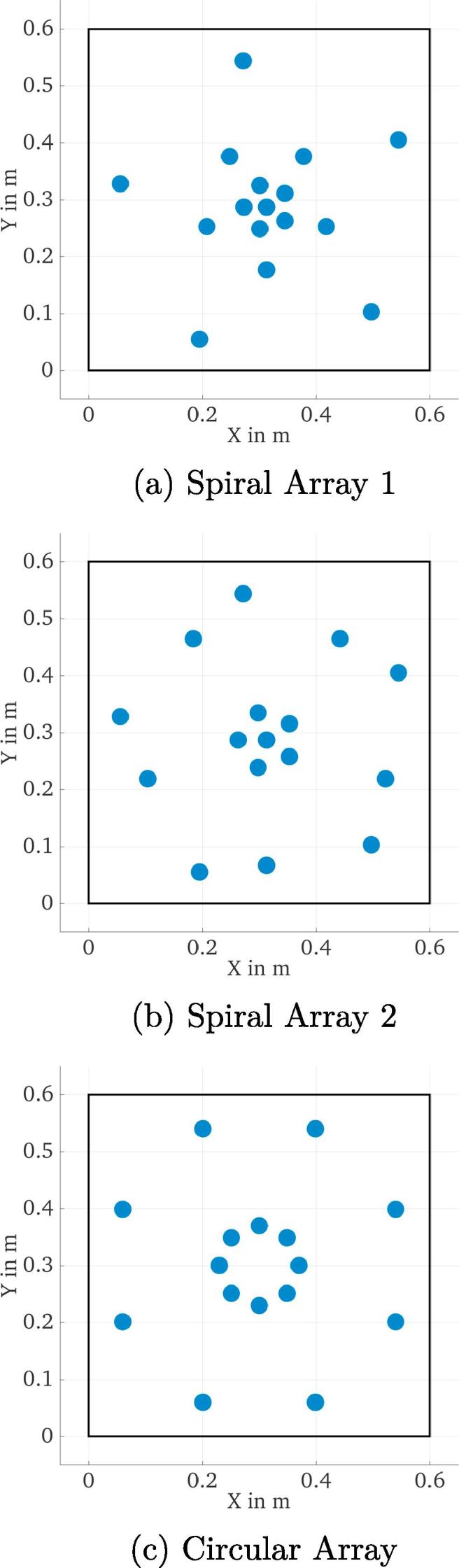
Three array geometries selected for optimization of shading weights, shown together with the ceiling tile boundaries (rectangle).

For a further analysis, three designs were selected: two versions of the logarithmic spiral array (Fig. 4 a and b) and a circular array (Fig. 4c). In the plots, the arrays are shown together with the boundary of a ceiling tile to indicate the design restrictions in terms of array size, where a safety margin of 4cm was left on all sides to prevent problems during the installation of the tiles. The two logarithmic arrays differ by the radii of the concentric circles: the array in Fig. 4a has more microphones concentrated in the center, whereas the one in Fig. 4b has two circles with larger radii and the rest of the microphones in the center.

Among the three selected designs, the array in Fig. 4b offers the best spatial resolution, due to the higher number of sensors with a larger radius. However, at the same time this also leads to the worst Maximum Sidelobe Level (MSL) among the three tested arrays. The logarithmic spiral in Fig. 4a and the circular array in Fig. 4c perform similarly, although the logarithmic spiral has the best MSL at high frequencies, whereas the circular array has a narrower beamwidth.

**Fig. 5:**
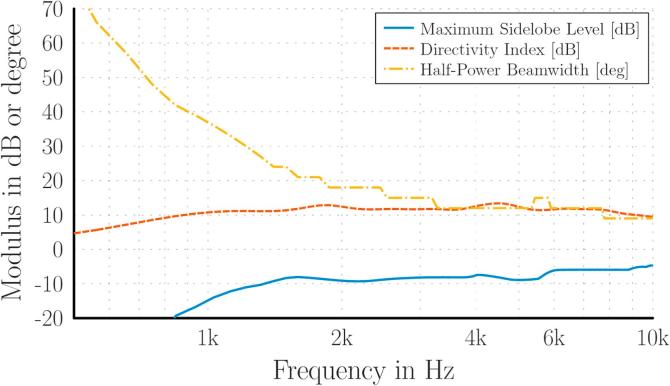
Array performance parameters of the array depicted in Fig. 4a, using frequency-dependent shading weights.

For the three designs chosen according to their broadband performance, the improvement achieved by using frequency-dependent shading weights for the individual circles of microphones in each array was analyzed. The operating range of the array (400–10,000 Hz) was chosen for this analysis. By choosing cut-on and cut-off frequencies for each of the circles of microphones, the effective outer diameter and number of sensors could be controlled and hence an optimal trade-off between the beamwidth and height of the sidelobes of the beampattern could be achieved. It was found that the exponential distribution of the array radii of the first logarithmic spiral array (Fig. 4a) is beneficial for frequency-dependent control of the beampattern. Also, since the logarithmic spiral array has one more circle of microphones compared to the circular array, this offers an additional degree of freedom for deriving the frequency-weights. Thus the logarithmic spiral array was chosen as the final layout. The design parameters for the ring radii as well as the shading weights are listed in Table 1. The resulting performance parameters are shown as a function of frequency in Fig. 5.

**Table 1:** Microphone ring radii and corner frequencies for the shading weights.

Radius	Cut-on Frequency	Cut-off Frequency
(in m)	(in kHz)	(in kHz)
0.0	2kHz	–
0.04	2kHz	–
0.11	–	–
0.26	–	4kHz

It can be seen that the shading weights provide an almost constant MSL (solid line in Fig. 5) and DI (dashed line) and a smooth increase in resolution towards higher frequencies, as can be seen from the decreasing HPBW values (dash-dotted line). This ensures an optimum performance in the operating frequency range under the given constraints.

**Fig. 6:**
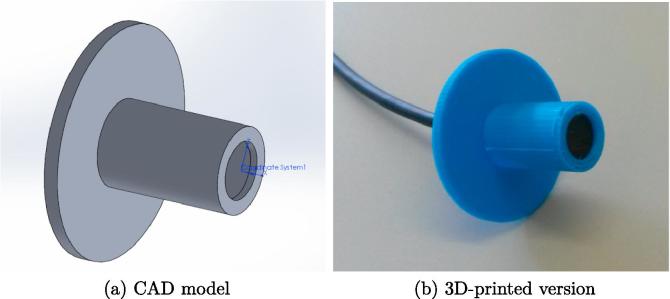
Microphone insert used to place microphones into the ceiling tile.

For the construction of the array, the microphone positions were marked on the ceiling tile by a laser-cutting machine to achieve the highest precision. The holes had to be cut by hand as the tile could not actually be cut by the laser. Nevertheless, it could be ensured for each 16-channel array that the positions were accurate within a tolerance of approximately 1mm. To mount the microphones, a 3D-printer was used to build inserts that enable a precise microphone placement and an easy exchange in case of malfunction. The CAD model and the actual print with a microphone inserted are shown in Fig. 6a and b, respectively.

## 4 Beamforming

With the calibrated audio data available from the array microphones, beamforming was used to determine the location of the most prominent acoustic sources. Different approaches to combining the microphone signals of the four array clusters were tested, such as independent beamforming using each cluster and the subsequent combination of the four outputs. However, the best results were achieved when all 64 microphones were used simultaneously as a single large array. For the processing steps and the results presented in the rest of this paper, this configuration was chosen.

To include early reflections in the beamforming processing, a relatively large block size of 8192 samples at a sampling rate of 48kHz (corresponding to a block length of 171ms) was chosen. Including early reflections could lead to improved spatial filtering results when a beamforming algorithm that takes advantage of source-coherence is used, as the sound from early reflections is coherent with the original source signal. This will be discussed further in Section 4.2.

A running estimate of the cross-spectral matrix $C_{n}(k)$ in the frequency domain is calculated based on the pressure data from all microphones $p_{n}(k)$ at time instant *n*, with a half-block overlap. Here, *k* is the wavenumber in rad/m. Using exponential smoothing, the estimate of the cross-spectral matrix at time instant *n* is

(1) $$C_{n}(k)=(1-\alpha )\;C_{n-1}(k)+\alpha \;p_{n}(k)p_{n}^{H}(k)\text{,}$$

where ${\left(\right)}^{H}$ is the complex conjugate transpose and α is the smoothing factor; its value was chosen here as 0.157, which corresponds to a filter decay time of 0.5s given the previously mentioned block size.

As most scan points are in the near-field of the array microphones, the steering vector for spherical wavefronts without distance compensation is used. An initial comparison showed that this method achieved an improved spatial filtering result compared to that obtained with a plane-wave steering vector, but this could be further explored for the installed system in a future study. For all beamforming calculations, the steering vector is thus calculated as (formulation I in [30]):

(2) $$w_{i}(k)=\frac{1}{M}{\text{e}}^{-\text{j}kd_{i}}\text{,}$$

where *M* is the number of microphones and $d_{i}$ is the vector of Euclidean distances between all microphones and the scan point with index *i*.

For this study, a total of 375 scan points on a rectangular grid at a height of 1.2m above the ground were chosen to cover the main area of the ICU with a 0.5m spacing. With a ceiling height of 2.6m, the distance between the plane of the array microphones and the scanning grid was 1.4m. The grid spacing of 0.5m was chosen as a compromise between calculation times and spatial resolution. An optimal value for the spacing of the scanning grid could be determined in a more detailed follow-up study.

Source localization is achieved by performing calculations on the scanning grid in three consecutive steps, which are described in the following subsections.

### 4.1 Delay-and-sum beamforming

In the first step, the beamforming output power $B_{n\text{,}i}(k)$ with conventional Delay-and-Sum beamforming is calculated in the operating frequency range for the audio block with index *n* and for each scanning point with index *i*:

(3) $$B_{n\text{,}i}(k)=w_{i}^{H}(k)C_{n}(k)w_{i}(k)\text{.}$$

As the continuous, real-time operation of the system is important, some considerations on the computational complexity were made to determine the best algorithm. It was found that the computation of the cross-spectral matrix (Eq. (1)) takes up most of the processing time, and hence a simpler version of Eq. (3) was also implemented:

(4) $$B_{n\text{,}i}(k)=(1-\alpha )\;B_{n-1\text{,}i}(k)+\alpha \;{\left|w_{i}^{H}(k)p_{n}(k)\right|}^{2}\text{.}$$

This version does not make use of the Cross-Spectral Matrix and hence no information about the cross-correlation between the microphones is available. It does however give exactly the same result as in Eq. (3), because the cross-correlation is not actually exploited in that algorithm. In comparison to Eq. (3), the implementation of Eq. (4) saves approximately 75% of the computation time.

**Fig. 7:**
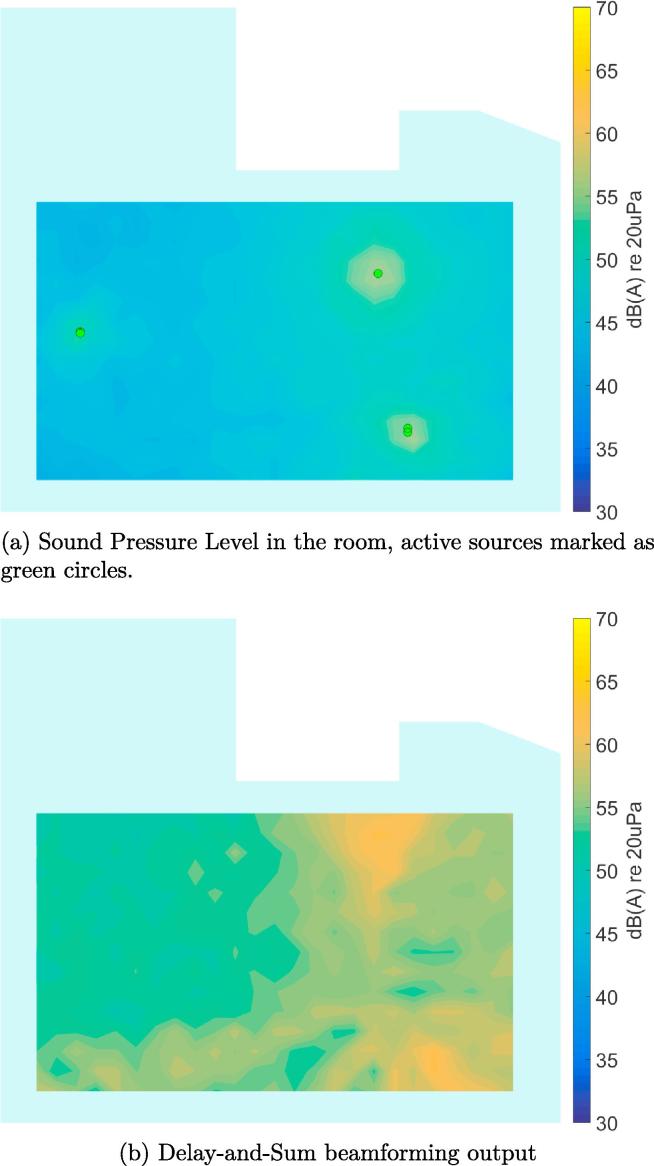
Processing example for a realistic simulation of the ICU environment (broadband levels are shown).

To give an idea of the processing output under ideal conditions, Fig. 7 shows an example for a simulation of the ICU environment, including actual recorded sounds and a simple room acoustics model. The model uses image sources [31] for the early reflections and adds statistical reverberation for the late reflections, using noise that is shaped to match the measured reverberation time in the ICU. In all of the following examples, the broadband, A-weighted sound pressure level (SPL) in dB(A)re20μPa is plotted. The broadband result for the simulated sound pressure magnitude $P_{n\text{,}i}(k)$ and the beamforming power output $B_{n\text{,}i}(k)$, respectively, is calculated for each scan point *i* as

(5) $$L_{P\text{,}n\text{,}i}=10\log_{10}\left(\frac{\sum_{k}w_{A}(k)P_{n\text{,}i}^{2}(k)}{p_{0}^{2}}\right)\text{,}$$

and

(6) $$L_{B\text{,}n\text{,}i}=10\log_{10}\left(\frac{\sum_{k}w_{A}(k)B_{n\text{,}i}(k)}{p_{0}^{2}}\right)\text{,}$$

where $p_{0}=20\;\mu \text{Pa}$ is the reference sound pressure and $w_{A}(k)$ is the squared magnitude of the A-weighting filter at wavenumber *k* [32].

In Fig. 7a the broadband SPL is plotted at a chosen time instant with three active sources. Please note that the location of these sources has been chosen as a hypothetical example to illustrate the processing steps and the source positions are not consistent with those in the following experimental results. Fig. 7b shows the broadband output level of the Delay-and-Sum beamformer. It becomes clear that this beamforming result cannot be used directly for an automatic, accurate source localization. The necessary post-processing steps are described in the following subsections.

### 4.2 Deconvolution (CLEAN/CLEAN-SC)

The result of the Delay-and-Sum beamformer for all scan points $B_{n}(k)$ can be regarded as the spatial convolution of the array response — including all sidelobes — with the source distribution $q_{n}(k)$. It is of course this source distribution that is of interest for localization and identification.

The deconvolution problem can be solved in several ways. The DAMAS algorithm is a direct deconvolution approach, which can be calculated with the Gauss-Seidel algorithm [33], or by enforcing sparseness in the result, e. g. with the FOCUSS algorithm [34]. However, both approaches are very computationally demanding, and so an iterative solution, such as the CLEAN [35] or CLEAN-SC algorithm [36] is usually favored. The iterative algorithms are faster than the direct approaches by two orders of magnitude, taking only approximately 25% more time than the Delay-and-Sum beamformer.

While the original CLEAN algorithm performs a straight-forward deconvolution based only on the array response, CLEAN-SC takes source correlation into account by working with the cross-spectral matrix. Hence, a trade-off between accuracy and computational demand can be made by the choice of the algorithm. An important advantage of CLEAN is that the array response can be pre-computed and thus processing time can be further reduced. For the application in the ICU, it has been found that contrary to expectations the inclusion of source coherence does not yield a significant improvement and hence the CLEAN algorithm with the fast Delay-and-Sum beamformer (Eq. (4)) is used. From this point on, only the CLEAN algorithm will be mentioned, but all processing is of course equally applicable to the CLEAN-SC version.

Regardless of the choice of CLEAN or CLEAN-SC, the result of this step of processing is a sparse beamforming map with few (typically less than 10) active sources for each frequency bin. When the spectral information is summed up to yield the broadband (A-weighted) level or other derived quantities, the map becomes slightly less sparse as the location of the sources in the scanning grid may not be absolutely stable across frequency. Nevertheless, the number of relevant sources is usually far less than the number of scan positions.

**Fig. 8:**
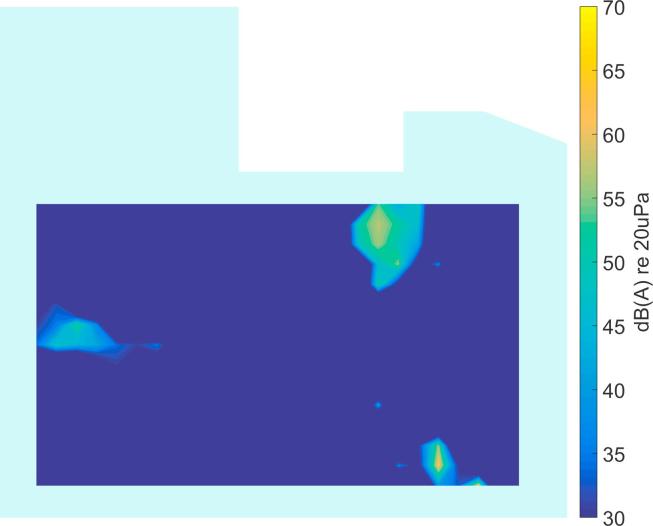
Broadband output level of the CLEAN algorithm for the simulated data in Fig. 7.

In Fig. 8 an example of the output from the CLEAN algorithm is shown for the simulated data in Fig. 7a. It can be seen that the main regions of source activity can be identified. However, some spurious locations also appear, probably from data in the low frequency range where the directivity of the arrays is not high, as demonstrated in Fig. 5. In the next subsection, a data clustering approach is described to overcome this problem.

### 4.3 Data clustering

The broadband result of the deconvolution — while relatively sparse — may not be directly useful to determine source locations automatically. This is a typical problem with beamforming results, which are usually interpreted by inspection of color maps per frequency, or frequency band. The goal of this project, however, is an automatic and autonomous localization of dominant sources, which could be used by a non-expert user, such as a member of the nursing staff.

To achieve this goal, the deconvolution results are combined using the *k-means* clustering algorithm [37]. The initialization is carried out with the *k-means++* algorithm [38]. In terms of input data for the clustering, two approaches were implemented and tested:

*Variant 1:* If only the broadband result of the CLEAN algorithm is available, the importance of each non-zero point in the map can only be determined from its level. The following steps are then taken:

1. Find non-zero elements and their indices in the broadband CLEAN beamforming map
2. Sort results according to level
3. Select the $N_{\max}$ highest level results
4. Extract the Cartesian coordinates corresponding to the indices determined in step 3
5. Perform *k-means* clustering while varying the maximum number of clusters as
  $$N_{\text{clusters}}=1\ldots \min(N_{\max}-1\text{,}5)$$
  and select the result with the maximum average silhouette value as the optimum [39]
6. Assign the sum of the powers of all points within each cluster as the effective cluster power

The maximum number of clusters is chosen to be equal to five as it is unlikely that there will be more than five active sources at the same time. Because the optimal number of clusters is determined automatically, the value of $N_{\max}$ is relatively unimportant, but here it is chosen to be 15.

The disadvantage of Variant 1 is that the only way to reject outliers, such as spurious peaks, is by level. Hence, if outliers with large levels appear in the beamforming map they will shift the cluster centroids and thus distort the result. A better approach is described in the following paragraph.

*Variant 2:* If information about the non-zero indices can be obtained for each frequency of interest, an additional pre-selection of the data can be performed in the following way:

1. Find all non-zero indices in the frequency range of interest
2. Sort the data according to the relative number of occurrences of each index across all frequencies
3. Select the indices that make up 80% of the data (based on the cumulative sum)
4. Continue with step 3 in Variant 1 above

The threshold of 80% was selected here as it yielded good results in terms of a reliable estimate of the number of active sources, but it is a parameter that can be adapted to the individual application.

**Fig. 9:**
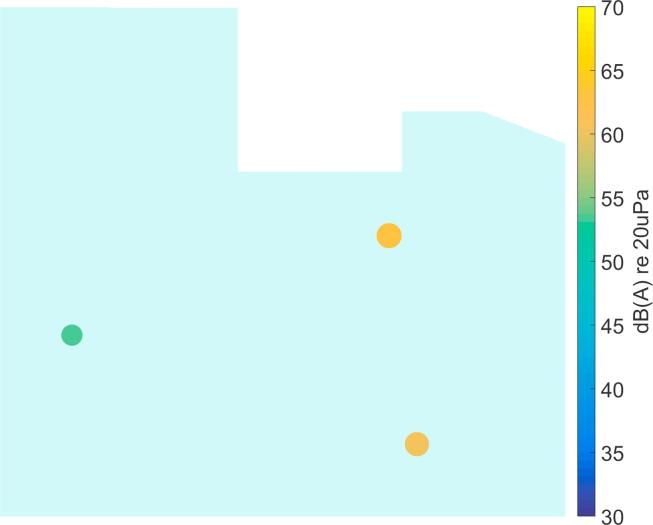
Clustering result of Variant 2 for the simulated data in Fig. 8, see Fig. 7 for the actual source locations.

By incorporating the number of occurrences across frequency, the data can be sorted according to importance and thus it is easier to reject outliers. The result of Variant 2 for the simulated data in Fig. 8 is presented in Fig. 9. Clearly, the relevant sources have been identified and outliers have been successfully suppressed. However, it should be said that in this example, the CLEAN result (Fig. 8) does not show too many spurious peaks, so in this case Variant 1 gives the same result as Variant 2. This is different in most real situations, as will be shown in the next section.

## 5 Results in the ICU

The array system described above was installed in the adult ward of the ICU at the John Radcliffe Hospital in Oxford at the end of October 2016. In this section, representative results are presented to give an idea of the actual system performance. Recordings were made during a typical shift with all beds in the ICU occupied. After applying the broadband calibration filters to the recorded data, the processing as described in Section 4 was applied.

It should be noted that the source locations identified from the recordings in the ICU (Fig. 10, Fig. 11, Fig. 12, Fig. 13, Fig. 14) are not expected to match the scenarios from the simulated examples (Fig. 7, Fig. 8, Fig. 9), as the simulations used purely hypothetical source locations.

**Fig. 10:**
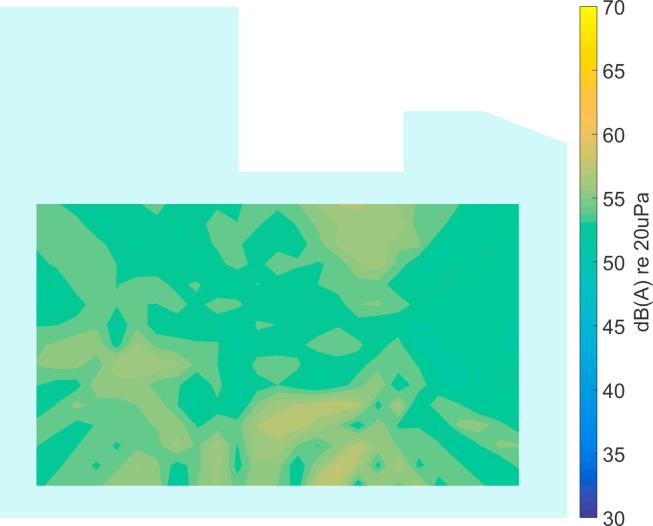
Processing result of Delay-and-Sum beamforming in the ICU environment (broadband levels are shown).

**Fig. 11:**
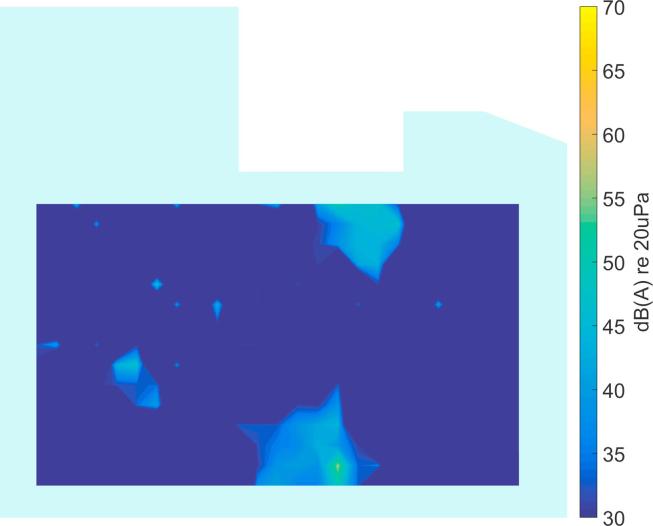
Broadband output level of the CLEAN algorithm for measured data in Fig. 10.

**Fig. 12:**
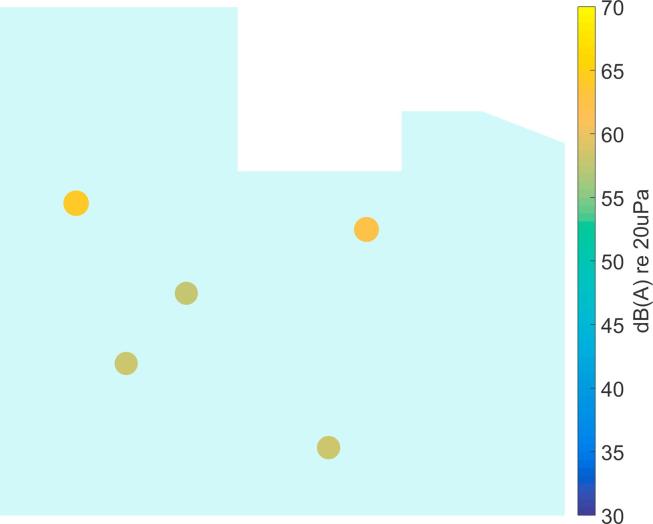
Clustering result of Variant 1 for the measured data in Fig. 11.

**Fig. 13:**
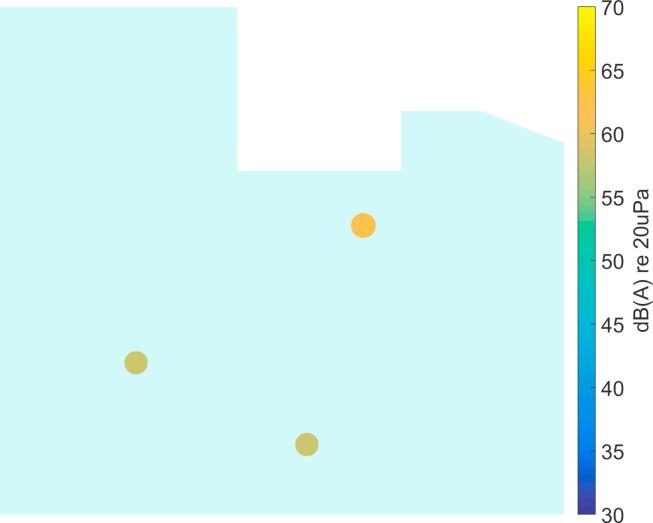
Clustering result of Variant 2 for the measured data in Fig. 11.

**Fig. 14:**
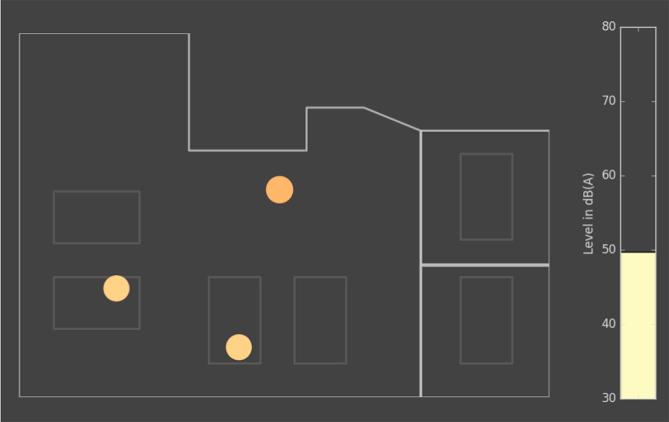
The data in Fig. 13 as it appears in the display for the nursing staff.

The beamforming map of the Delay-and-Sum algorithm at one chosen time instant is shown in Fig. 10. As already observed for the simulated data in Section 4.1, it becomes clear that this result cannot be directly used for source localization.

In Fig. 11 the deconvolution result of the CLEAN algorithm is presented, yielding a much sparser map that shows the areas with dominant sources. In comparison to the simulated data in Section 4.2 (Fig. 8), the measured data shows more spurious peaks, which may be due to additional sources, or may be caused by array imperfections. It is precisely this behavior that makes it necessary to further process the data before an automatic localization can be achieved.

The deconvolution output is processed with Variant 1 of the clustering algorithm (Section 4.3) and the result is shown in Fig. 12. In comparison, the result of Variant 2 of the clustering algorithm is shown in Fig. 13 and from this results it can be seen that by only taking into account the level information, more sources are detected than are actually present. This indicates the benefit of using the additional information across frequency, as is done in Variant 2.

It seems that the relevant sources were correctly localized by Variant 2 of the clustering approach. However, it should be stressed here that the measurements were performed in a working ICU environment, which could not be controlled, so the precise number and location of active sources was not known. Further studies under controlled conditions will have to be carried out to establish the robustness of the presented approach and the ideal set of parameters.

The final display for the nursing staff, implemented in the Julia programming language [40], is presented in Fig. 14. In addition to the source locations, the average sound pressure level recorded by the microphones is shown in a bar graph on the right side of the display. The positions of the detected sources are also exported continuously, so that a statistical evaluation over time is possible.

## 6 Conclusions

In this paper a microphone array system has been presented that is used to remotely localize and quantify acoustic noise sources in an Intensive Care Unit. The hardware and array design for the system under the given constraints have been described. An alternative beamforming formulation resulting in faster computation has been developed and a clustering approach has been introduced to enable an automatic localization of the most dominant acoustic sources.

It has been shown that the raw result of beamforming calculations is not directly useful for unsupervised localization. While the deconvolution approach to beamforming results in a much sparser source map, spurious peaks can still influence and degrade the localization performance. The automatic clustering algorithm has been implemented in the array system. With the help of clustering, the influence of spurious peaks in the source map can be effectively suppressed. The successful application of the presented approach has been demonstrated through real measured data from the running system.

Further studies will be necessary to confirm the validity and robustness of the automatic localization approach, as the data gathered so far was not obtained under controlled laboratory conditions. The optimum number of clusters and its dependence on the environment and the number of array sensors will have to be investigated.

The system described here is currently used in the John Radcliffe Hospital in Oxford to obtain a better impression of the acoustic source distribution in the ICU. This information will be used to devise measures to provide an overall reduction in background noise levels, hopefully leading to improved patient recovery. At the moment, the possibility of classifying individual sources by combining the spatial filtering presented here with machine learning is being investigated. This additional information would enable the nursing staff to, for example, distinguish between medical equipment and speech, making it easier to implement changes to reduce the noise level.

Before the system described in this paper can be deployed in additional ICUs or similar healthcare environments, its effectiveness and influence on staff and patients will have to be determined. This will require a long-term study, which will then hopefully establish whether the array-based system can assist medical staff in reducing the ICU noise levels.
